# Paradoxical Role of Prion Protein Aggregates in Redox-Iron Induced Toxicity

**DOI:** 10.1371/journal.pone.0011420

**Published:** 2010-07-06

**Authors:** Dola Das, Xiu Luo, Ajay Singh, Yaping Gu, Soumya Ghosh, Chinmay K. Mukhopadhyay, Shu G. Chen, Man-Sun Sy, Qingzhong Kong, Neena Singh

**Affiliations:** 1 Department of Pathology, Case Western Reserve University, Cleveland, Ohio, United States of America; 2 Special Center for Molecular Medicine, Jawaharlal Nehru University, New Delhi, India; Ohio State University, United States of America

## Abstract

**Background:**

Imbalance of iron homeostasis has been reported in sporadic Creutzfeldt-Jakob-disease (sCJD) affected human and scrapie infected animal brains, but the contribution of this phenotype to disease associated neurotoxicity is unclear.

**Methodology/Principal Findings:**

Using cell models of familial prion disorders, we demonstrate that exposure of cells expressing normal prion protein (PrP^C^) or mutant PrP forms to a source of redox-iron induces aggregation of PrP^C^ and specific mutant PrP forms. Initially this response is cytoprotective, but becomes increasingly toxic with time due to accumulation of PrP-ferritin aggregates. Mutant PrP forms that do not aggregate are not cytoprotective, and cells show signs of acute toxicity. Intracellular PrP-ferritin aggregates induce the expression of LC3-II, indicating stimulation of autophagy in these cells. Similar observations are noted in sCJD and scrapie infected hamster brains, lending credence to these results. Furthermore, phagocytosis of PrP-ferritin aggregates by astrocytes is cytoprotective, while culture in astrocyte conditioned medium (CM) shows no measurable effect. Exposure to H_2_O_2_, on the other hand, does not cause aggregation of PrP, and cells show acute toxicity that is alleviated by CM.

**Conclusions/Significance:**

These observations suggest that aggregation of PrP in response to redox-iron is cytoprotective. However, subsequent co-aggregation of PrP with ferritin induces intracellular toxicity unless the aggregates are degraded by autophagosomes or phagocytosed by adjacent scavenger cells. H_2_O_2_, on the other hand, does not cause aggregation of PrP, and induces toxicity through extra-cellular free radicals. Together with previous observations demonstrating imbalance of iron homeostasis in prion disease affected brains, these observations provide insight into the mechanism of neurotoxicity by redox-iron, and the role of PrP in this process.

## Introduction

Prion disorders are a group of neurodegenerative conditions of humans and animals that are sporadic, inherited, and infectious in nature. The main pathogenic event in all prion disorders is change in conformation of a normal cell surface glycoprotein, the prion protein (PrP^C^), to a β-sheet rich isoform referred to as PrP-scrapie (PrP^Sc^) [1]. Most human prion disorders are sporadic in nature, and are initiated by conversion of PrP^C^ to PrP^Sc^ by a stochastic event. Sporadic Creutzfeldt-Jakob disease (sCJD) is a typical example [2]–[4]. Inherited forms comprise 10–15% of all cases, and are associated with point mutations in the prion protein gene (*PRNP*) [5]. Infectious disorders are relatively rare, and are acquired when an exogenous source of PrP^Sc^ induces a change in conformation of host PrP^C^ to PrP^Sc^ as in variant CJD of humans, scrapie of sheep, and chronic wasting disease of the deer and elk population [6]–[8]. For all prion disorders, manifestation of disease requires the expression of host PrP^C^ on neuronal plasma membrane, where it provides the necessary substrate for PrP^Sc^ and facilitates transmission of the neurotoxic signal [9], [10]. Although our understanding of events underlying the conversion of PrP^C^ to PrP^Sc^ and mechanism(s) of neurotoxicity by PrP^Sc^ has improved significantly over the past years, specific nature of the toxic signal and the role of PrP^C^ in transmitting this signal are still unclear.

Several mechanisms of toxicity by PrP^Sc^ have been suggested. Principal among these are loss of normal function of PrP^C^ due to aggregation, and gain of toxic function by PrP^Sc^ that requires plasma membrane expression of PrP^C^ to be effective [11], [12]. Among triggers that induce aggregation of PrP^C^, redox-active metals such as copper and iron are of particular interest since PrP^C^ is involved in their metabolism, and aggregation of PrP^C^ to the PrP^Sc^ form is likely to alter their homeostasis in affected brains [13]–[20]. In support of this hypothesis, scrapie infected hamster brains show increased imbalance of iron homeostasis with disease progression, and prion disease affected human and mouse brains accumulate Fe^2+^ and Fe^3+^ ions, some in association with PrP^Sc^ deposits [19], [21]–[24]. In neuroblastoma cells redox-iron induces aggregation of PrP^C^ to a PrP^Sc^-like form that co-aggregates with the iron storage protein ferritin, partly explaining the underlying cause of iron imbalance in diseased brains [25].

To understand the relationship between redox-iron, PrP^C^, PrP^Sc^, and associated cytotoxicity, we used cells expressing PrP^C^ and mutant forms of PrP as models. Comparison with an alternative source of free radicals such as H_2_O_2_ provided additional information on the underlying mechanism of toxicity by free radicals. We chose this approach since familial prion disorders are likely to provide important mechanistic insight into the more common but difficult to model sporadic disorders. Moreover, cell models offer the simplicity, sensitivity, and specificity of read out that is often difficult to achieve in the complex milieu of the brain. Where possible, parallels are drawn with scrapie infected cell lines, hamster brains, and sCJD affected human brain tissue to validate the results from cell models. We report that PrP functions as a sink for redox-iron induced free radicals but not H_2_O_2_ by undergoing aggregation, thereby protecting cells from toxicity. Mutations in PrP influence redox-iron induced PrP aggregation and cytotoxicity differentially, providing information on the role of PrP in this process. In addition, autophagy and astrocyte mediated phagocytosis reduce redox-iron induced toxicity, demonstrating the complexity of different biochemical processes and cell types in determining prion disease associated neuronal degeneration.

## Results

### Exogenous redox-iron induces aggregation of PrP^C^ and selective mutant PrP forms

Exposure of PrP^C^-expressing human neuroblastoma cells to a source of redox-iron induces co-aggregation of PrP^C^ and cellular ferritin within lysosomes [25]. To evaluate whether mutations within the coding sequence of PrP influence this response, neuroblastoma cells expressing PrP^C^ and mutant forms PrP^P105L^ or PrP^P102L^ that segregate with GSS, and PrP^Δ51-89^ or PrP^Δ23-89^ lacking the copper binding octa-peptide repeat sequence or N-terminal 90 amino acids respectively were exposed to 0.1 mM ferric ammonium citrate (FAC) or PBS for 48 hours and subjected to differential centrifugation to identify aggregated PrP forms. This procedure involves centrifugation of lysates at 290 g to isolate low speed detergent soluble supernatant (S1) and insoluble pellet fractions (P1), followed by ultra-centrifugation of S1 at 100,000 g to separate high speed detergent soluble (S2) and insoluble (P2) fractions. Under normal conditions, majority of PrP^C^ and ferritin fractionate in the detergent soluble S1 and S2 fractions. Only minimal amounts are detected in the high speed pellet fraction P2.

**Figure 1:**
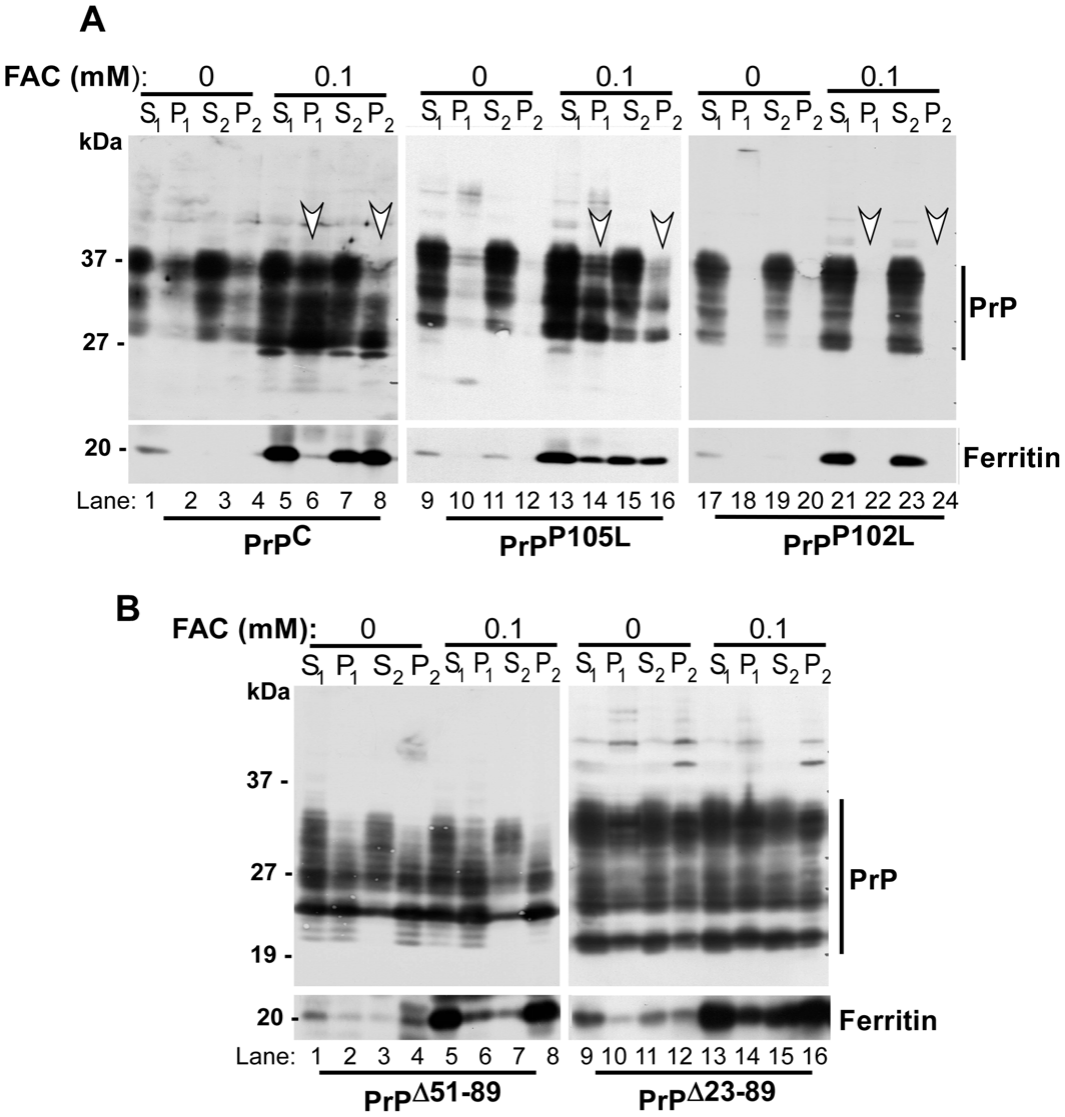
Exogenous redox-iron induces aggregation of PrP^C^ and certain mutant PrP forms. (**A and B**) Control and FAC exposed lysates from PrP^C^, PrP^105L^, PrP^102L^, PrP^Δ51-89^, and PrP^Δ23-89^ cells were subjected to differential centrifugation, and fractionated proteins were probed for PrP and ferritin. A significant amount of PrP^C^ and PrP^105L^ partition in the detergent insoluble P1 and P2 fractions following exposure to FAC (panel A, lanes 1–4 vs. 5–8 and lanes 9–12 vs. 13–16), while PrP^102L^ partitions mainly in the detergent soluble S1 and S2 fractions (panel A, lanes 17–24), and PrP^Δ51-89^ and PrP^Δ23-89^ partition equally between soluble (S1, S2) and insoluble (P1, P2) fractions (panel B, lanes 1–16) in the absence or presence of FAC.

As expected, PrP^C^ from control cells is detected in S1 and S2 fractions (Figure 1 A, lanes 1 and 3). Minimal amounts partition in the low and high speed pellet fractions P1 and P2 (Figure 1 A, lanes 2 and 4). Following a 48 hours exposure to FAC, PrP^C^ is up regulated (Figure 1 A, lane 1 vs. 5), and a significant amount partitions in the detergent insoluble P1 and P2 fractions (Figure 1 A, lanes 6 and 8) [25]. A similar evaluation of PrP^P105L^-cells reveals mostly detergent soluble forms of PrP^105L^ in control cells (Figure 1 A, lanes 9 and 11). Exposure to FAC causes up regulation as observed for PrP^C^ (Figure 1 A, lane 9 vs. 13), and re-distribution of PrP^105L^ to detergent insoluble fractions P1 and P2 (Figure 1 A, lanes 14 and 16). PrP^P102L^ is also up regulated in response to FAC (Figure 1 A, lane 17 vs. 21), but unlike PrP^C^ and PrP^P105L^, partitions entirely in the detergent soluble S1 and S2 fractions regardless of exposure to FAC (Figure 1 A, lanes 21–24). PrP^Δ51-89^ and PrP^Δ23-89^ are not up regulated by FAC (Figure 1 B, lanes 1 vs. 5 and 9 vs. 13), and partition equally between detergent soluble and insoluble fractions in the absence or presence of FAC (Figure 1 B, lanes 1–16).

Re-probing for ferritin shows significant up regulation following exposure to FAC, and variable distribution between detergent soluble and insoluble fractions in the cell lines tested (Figures 1 A and B). The only exception is cells expressing PrP^P102L^, where ferritin partitions exclusively in S1 and S2 fractions (Figure 1 A, lanes 17, 19, 21 and 23). Staining of PVDF membranes with Ponceu-S for all protein bands shows that the observed differences in PrP distribution are not an artifact of protein loading (Figure S1 A and S1 B).

**Figure 2:**
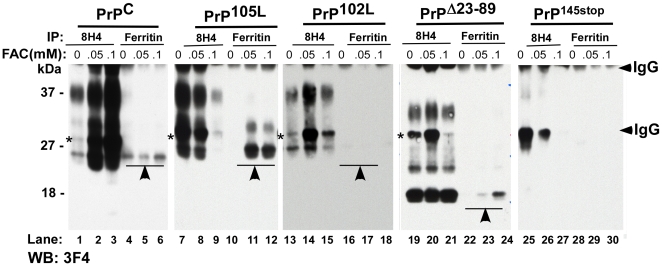
PrP and ferritin co-immunoprecipitate in FAC treated cells. Cell lysates prepared from PrP^C^, PrP^105L^, PrP^102L^, PrP^Δ23–89^, and PrP^145stop^-cells exposed to the indicated concentrations of FAC were immunoprecipitated with either anti-PrP antibody 8H4 or anti-ferritin antibody, and eluted proteins were immunoblotted with anti-PrP antibody 3F4. Immunoblotting of 8H4 immunoprecipitates reveals the expected glycoforms of PrP for all cell lines as expected (lanes 1–3, 7–9, 13–15, 19–21, and 25–27). Antibody to ferritin co-immunoprecipitates unglycosylated form of PrP^C^ (lanes 4–6), unglycosylated and diglycosylated forms of PrP^105L^ (lanes 10–12), minimal amounts of unglycosylated PrP^102L^ (lanes 16–18), unglycosylated PrP^Δ23–89^ (lanes 22–24), and no PrP forms from PrP^145stop^-cells (lanes 28–30). Co-immunoprecipitated PrP forms increase with increasing concentration of FAC (lanes 4 vs. 6, 10 vs. 12, and 22 vs. 24). A small amount of PrP^C^ co-immunoprecipitates with ferritin even in the absence of FAC (lane 4).

To evaluate whether PrP and ferritin form a complex, PrP^C^, PrP^105L^, PrP^102L^, PrP^Δ23–89^, and PrP^145stop^-cells were exposed to 0, 0.05 and 0.1 mM of FAC for 48 hours, and clarified lysates were immunoprecipitated with either anti-PrP (8H4) or anti-ferritin antibody followed by immunoblotting of eluted proteins with a different anti-PrP antibody 3F4. Eluates from all cell lines show the expected glycoforms of PrP in 8H4 immunoprecipitates (IP) except the PrP^145stop^ sample that lacks the epitope for 8H4 (Figure 2 A, lanes 1–3, 7–9, 13–15, 19–21, and 25–27). Immunoprecipitation with anti-ferritin antibody, on the other hand, co-precipitates unglycosylated form of PrP^C^ (lanes 4–6), unglycosylated and diglycosylated forms of PrP^105L^ (lanes 10–12), barely detectable unglycosylated PrP^102L^ (lanes 16–18), unglycosylated PrP^Δ23–89^ (lanes 22–24), and no PrP^145stop^ (lanes 28–30). The amount of PrP co-precipitating with anti-ferritin appears to increase on exposure of cells to FAC, though it is difficult to draw quantitative comparisons from IP results. The observed results are not due to non-specific binding of PrP or ferritin to beads or antibody since no protein bands are detected in the PrP^145stop^ sample (Figure 2 A, lanes 25–30). Together, these observations suggest that PrP and ferritin form a complex, especially after exposure to FAC.

### PrP co-aggregates with ferritin in lysosomes and autophagosomes

**Figure 3:**
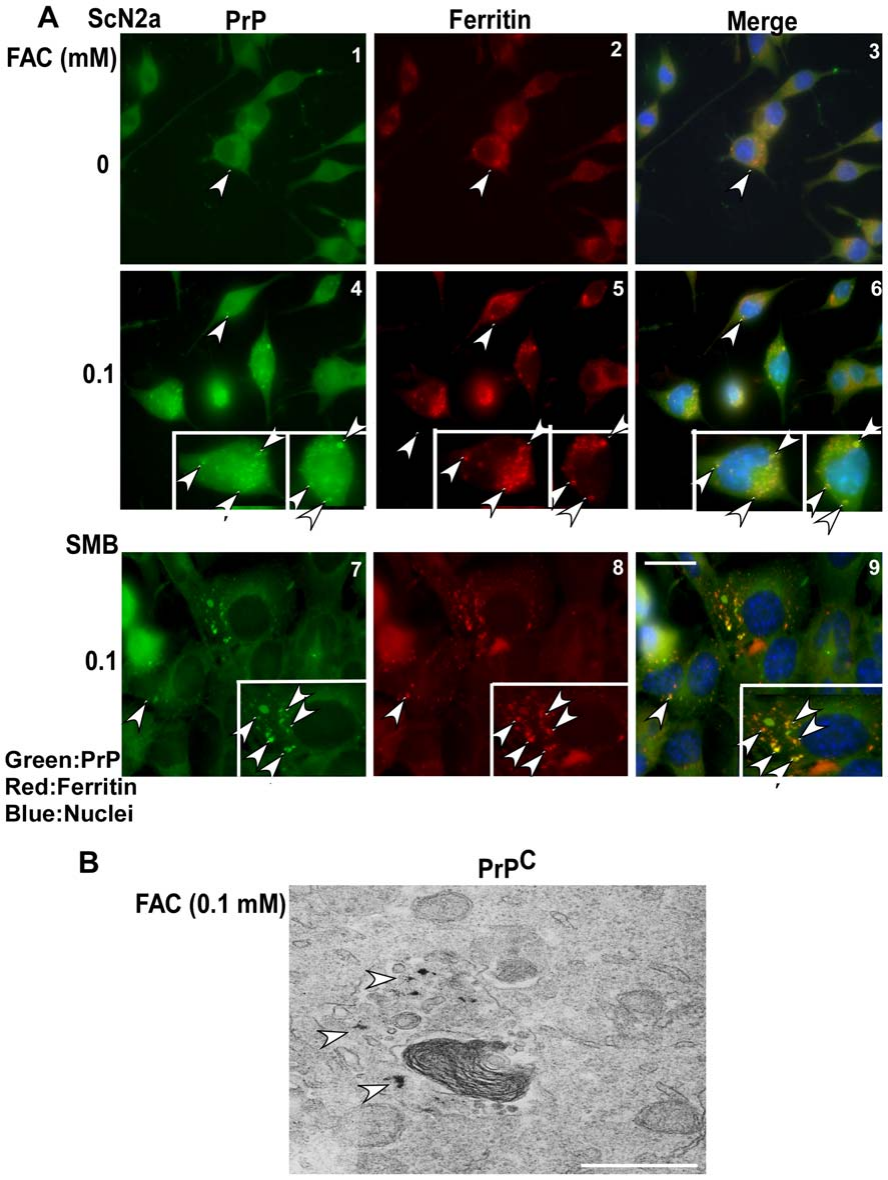
PrP-ferritin aggregates accumulate lysosomes and autophagosomes. (**A**) ScN2a and SMB cells cultured in the presence of 0.1 mM FAC or vehicle were permeabilized and immunoreacted with PrP specific mouse monoclonal 8H4 followed by anti-mouse FITC, and polyclonal anti-ferritin followed by anti-rabbit-TRITC antibodies. FAC exposed cells show prominent aggregates of PrP and ferritin in membrane-enclosed structures (panels 4–9). Control cells also show similar aggregates, but the amount is several-fold less than treated cells (panels 1–3). Bar: 10 µm. (**B**) Electron-microscopy of FAC exposed PrP^C^-cells shows electron-dense iron deposits in structures consistent with lysosomes and/or autophagosomes. Bar: 1 µm.

Relevance of the above observations to prion disease pathogenesis was assessed by performing a similar analysis on scrapie infected cell lines ScN2a and SMB. Exposure of cells to 0.1 mM FAC for 24 hours followed by immunostaining for PrP using 8H4-anti-mouse-FITC (green) and anti-ferritin-anti-rabbit-TRITC (red) shows co-immunostaining of PrP and ferritin in vesicular structures consistent with lysosomes (Figure 3 A, panels 1–3, arrow-heads). Exposure to FAC increases the expression and co-localization of PrP and ferritin significantly in both cell lines as noted in Figure 1 A above and previous reports (Figure 3 A, panels 4–9, arrow-heads) [25], [26].

The cellular compartment(s) of ferritin accumulation was further characterized by processing FAC exposed PrP^C^-cells for electron microscopy (EM) to visualize iron rich and thereby electron-dense ferritin aggregates. Dark granular deposits of iron are clearly visible in single-membrane enclosed lysosomes containing lamellar structures and cell debris (Figure 3 B, arrow-heads). Control cells cultured in normal medium do not show similar deposits (data not shown).

**Figure 4:**
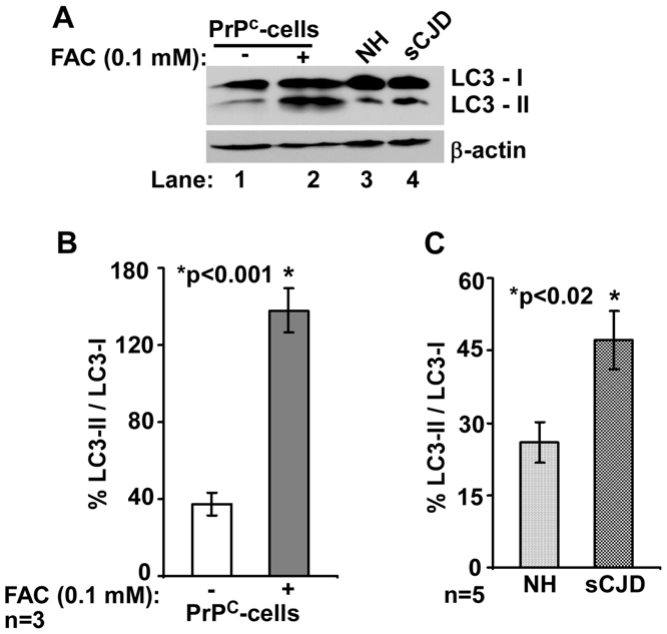
Autophagy is up regulated in FAC exposed cells and sCJD brains. (**A**) Control and FAC exposed PrP^C^ cell lysates and brain homogenates prepared from sCJD and age-matched controls were analyzed by Western blotting and probed with anti-LC3 antibody and re-probed for β-actin. FAC exposed lysates and sCJD samples show higher levels of LC3-II relative to controls (lanes 1 vs. 2 and 3 vs. 4). (**B and C**) Quantitative estimation shows significant increase in LC3-II vs. LC3-I in FAC treated lysates and sCJD samples relative to matched controls.

**Figure 5:**
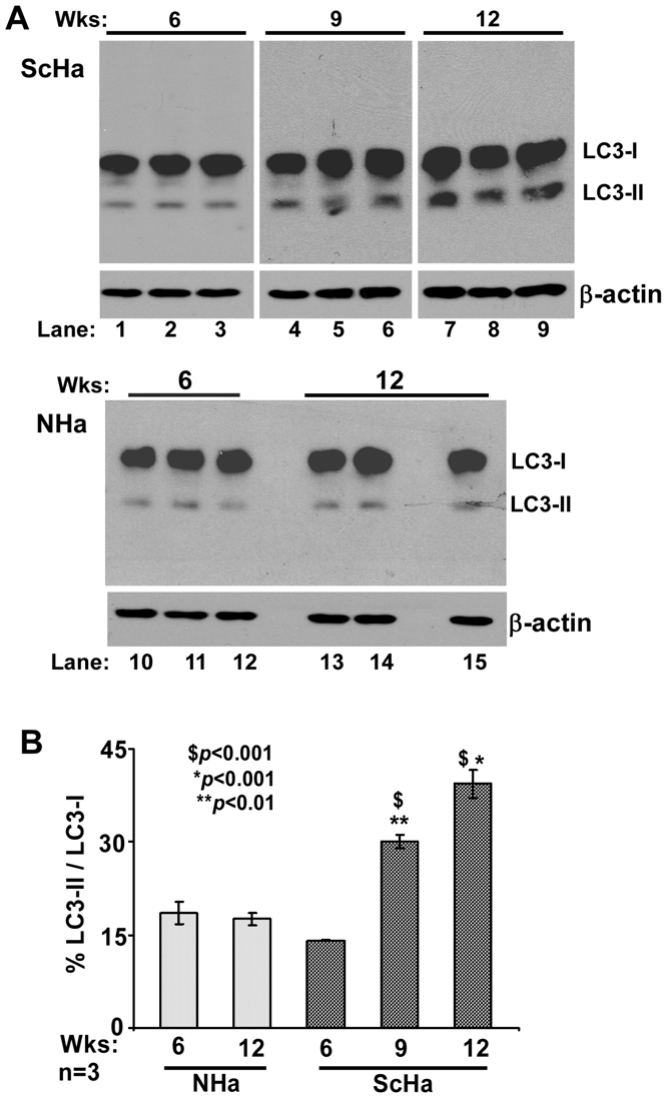
Autophagosomes increase with prion disease progression. (**A**) Immunoblotting of scrapie infected hamster brains with LC3 antibody shows increase in LC3-II levels with disease progression relative to age-matched controls (lanes 1–9 vs. 10–15). (**B**) Quantitative estimation after normalization with β-actin shows significant increase in LC3-II relative to LC3-I in diseased samples 9 and 12 weeks post-inoculation relative to matched controls. *p<0.001; **p<0.01 as compared to NHa. In addition, diseased samples show a significant increase in LC3-II in 9 and 12 weeks post-inoculation samples relative to the 6 week sample. **^$^** *p*<0.001 relative to ScHa at 6 weeks.

To evaluate whether these compartments represent autophagosomes, lysates prepared from control and FAC exposed cells and brain homogenates from sCJD and age-matched controls were immunoblotted for LC3-I and II (Figure 4 A, lanes 1–4) [27]–[29]. FAC treated lysates show an increase in LC3-II by 3.7 fold relative to untreated controls (Figure 4 B), and sCJD homogenates show a 1.8 fold increase compared to age-matched controls (Figure 4 C). A similar analysis of scrapie infected hamster brains shows higher levels of LC3-II at 12 weeks post-inoculation (pi) relative to matched controls (Figure 5 A, lanes 7–9 vs. 13–15). Furthermore, LC3-II levels increase as the disease progresses from 6 to 12 weeks pi (Figure 5 A, lanes 1–9), while matched controls show minimal change (Figure 5 A, lanes 10–15). Quantitative estimation of LC3-II vs. LC3-I after normalization with β-actin shows an increase of 1.6 and 2.2 fold at 9 and 12 weeks pi relative to 12 week controls (Figure 5 B), and an increase of 1.8 and 2.4 fold at 9 and 12 weeks pi relative to 6 weeks pi (Figure 5 B). These observations indicate up regulation of autophagy on exposure of cells to FAC, in sCJD brains, and in scrapie infected hamster brains with disease progression.

### PrP modulates iron induced cytotoxicity

**Figure 6:**
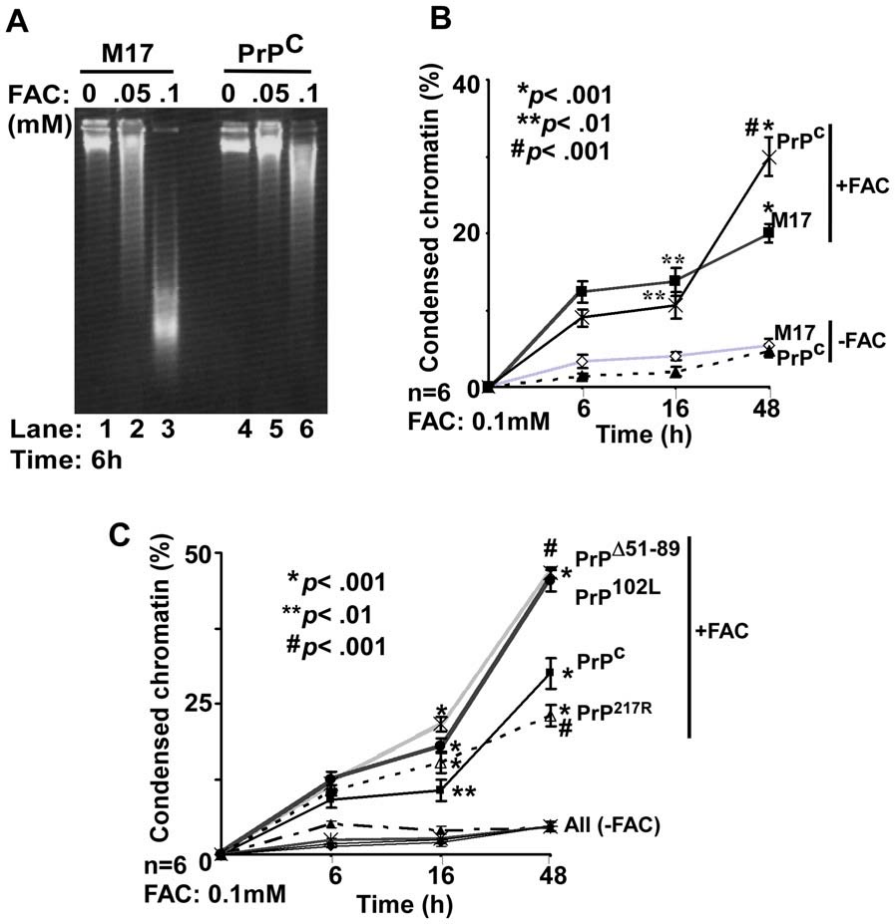
PrP modulates FAC induced cytotoxicity. (**A**) M17 and PrP^C^-cells were exposed to indicated concentrations of FAC and extracted DNA was visualized. Exposure to 0.1 mM FAC causes fragmentation of DNA from M17 cells and minimal change in the PrP^C^ sample (lanes 3 vs. 6). (**B**) Quantification of FAC induced toxicity at the indicated times shows significant resistance of PrP^C^-cells after 6 and 16 hours, and an exponential increase after 48 hours relative to M17 cells. *p<0.001; **p<0.01 as compared to untreated controls; **^#^** *p*<0.001 as compared to 16 hour FAC treated PrP^C^ cells. (**C**) Comparison of mutant cell lines with PrP^C^-cells shows significantly higher toxicity of PrP^102L^ and PrP^Δ51–89^-cells at 16 hours and 48 hours relative to PrP^C^-cells. PrP^217R^-cells show increased toxicity after 16 hours followed by a decline in cell death after 48 hours. *p<0.001; **p<0.01 as compared to untreated controls; **^#^** *p*<0.001 as compared to 48 hour FAC treated PrP^C^ cells.

The role of PrP^C^ in redox-iron induced toxicity was evaluated by exposing M17 cells expressing low levels of PrP or transfected to express 6–10 fold higher levels of PrP^C^ and mutant PrP forms to FAC (Figures 6–8 and Table 1). Exposure to 0.05 or 0.1 mM FAC for 6 hours followed by DNA fragmentation assay shows higher sensitivity of M17 relative to PrP^C^-cells (Figure 6 A, lanes 3 and 6). Evaluation of cell viability at different time points following exposure to FAC shows relatively less cytotoxicity in PrP^C^-cells after 6 and 16 hours, and a surprising 50% increase after 48 hours of exposure relative to M17 cells (Figure 6 B). Control M17 and PrP^C^ cells cultured in the absence of FAC show minimal differences in viability for up to 48 hours (Figure 6 B). A similar evaluation of mutant cells lines with PrP^C^-cells shows an increase in cytotoxicity by 37, 69, and 51% in PrP^102L^-cells, and 29, 100, and 55% in PrP^Δ51–89^-cells after 6, 16, and 48 hours of exposure respectively (Figure 6 C). Surprisingly, PrP^217R^ cells show higher number of dead cells at 6 and 16 hours, and a decrease by 23% relative to PrP^C^-cells after 48 hours of exposure (Figure 6 C). Control PrP^C^ and mutant cell lines show minimal cytotoxicity at all time points tested (Figure 6 C). Thus, PrP^C^-cells show relative resistance to FAC at early time points, and an exponential increase in cell death at later times. Mutant cell lines PrP^102L^ and PrP^Δ51–89^ show higher sensitivity relative to PrP^C^-cells at all time points, while PrP^217R^-cells show increased sensitivity at early time points and relative resistance after 48 hours of exposure (Figure 6 C).

**Figure 7 pone-0011420-g007:**
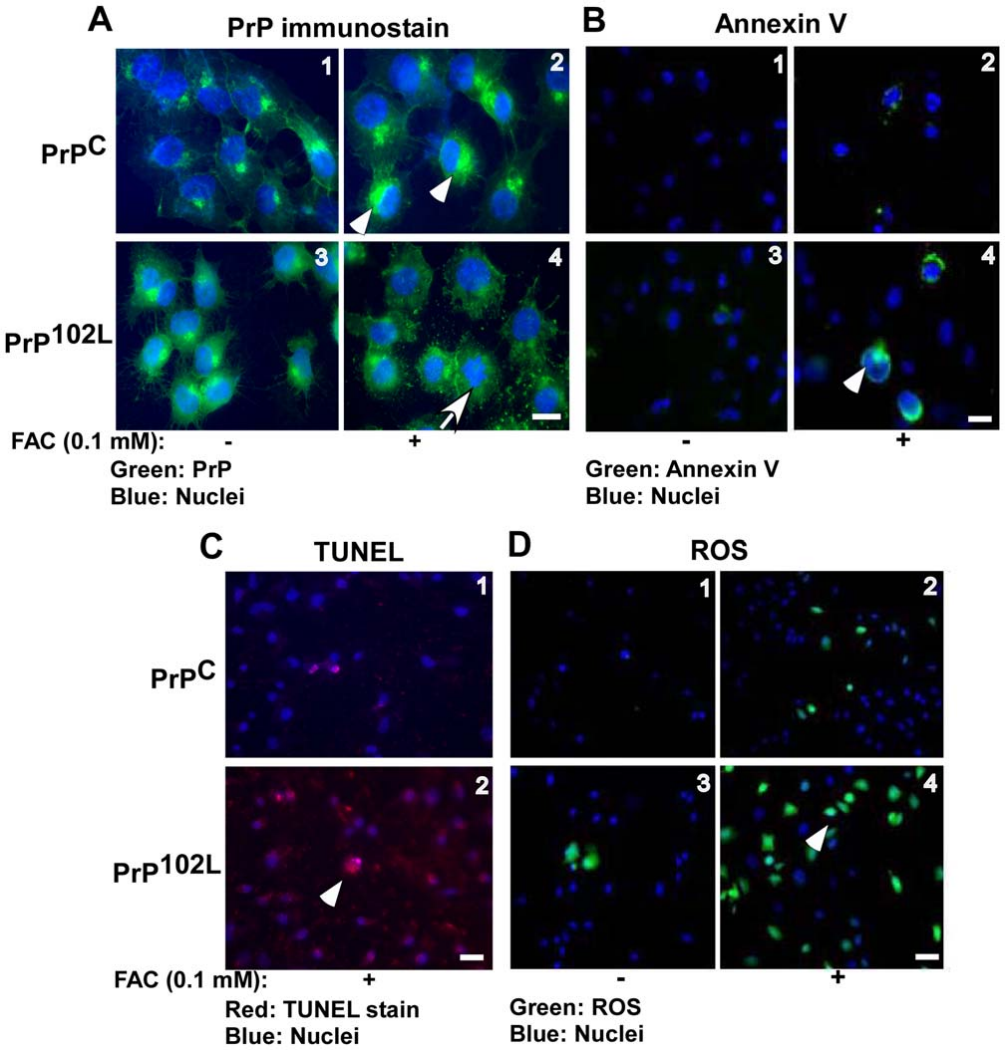
Aggregation of PrP is cytoprotective. (**A**) Immunoreaction with 8H4 shows intracellular aggregates of PrP^C^ following exposure to FAC (panel 2), and minimal aggregation of PrP^102L^ under similar conditions (panel 4). Bar: 10 µm. (**B**) Reaction with Annexin V shows minimal effect of FAC on PrP^C^-cells (panels 1 and 2), while PrP^102L^-cells show increased reaction (panels 3 and 4). Bar: 10 µm. (**C**) FAC exposure increases the number of TUNEL positive PrP^102L^-cells significantly relative to PrP^C^-cells (panels 1 and 2). Bar: 10 µm. (**D**) PrP^102L^-cells show significantly more reaction for ROS after exposure to FAC compared to similarly treated PrP^C^-cells (compare panels 2 and 4). Bar: 10 µm.

**Figure 8:**
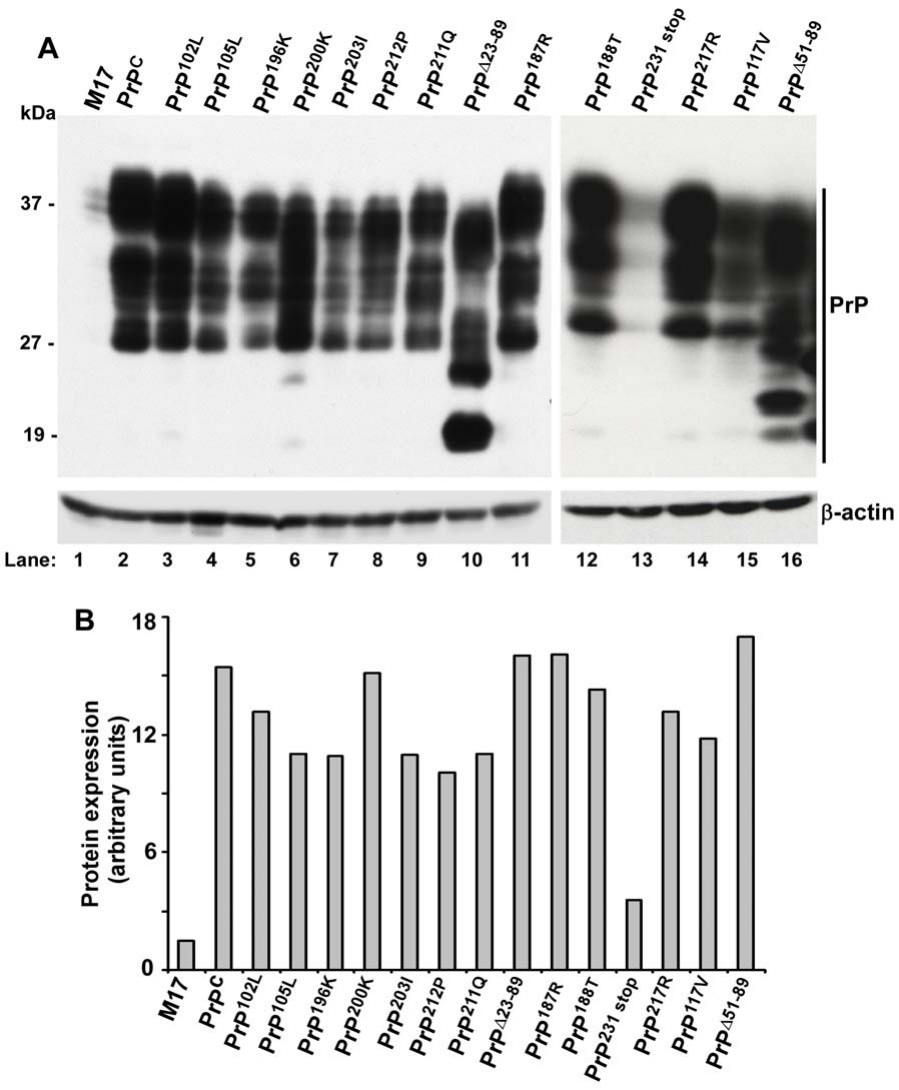
Cytoprotective effect of PrP is independent of its expression level. (**A**) Equal amount of protein from lysates of M17, PrP^C^, PrP^102L^, PrP^105L^, PrP^196K^, PrP^200K^, PrP^203I^, PrP^212P^, PrP^211Q^, PrP^Δ23–89^, PrP^187R^, PrP^188T^, PrP^231stop^, PrP^217R^, PrP^117V^, and PrP^Δ51–89^ was subjected to Western blotting. Probing for PrP shows the expected glycoforms of PrP in all cell lines (lanes 1–16). Re-probing for β-actin shows equal loading of protein. (**B**) Quantification after normalization with β-actin shows 6–10 fold higher expression of PrP^C^ and mutant cell lines relative to M17 cells. The only exception are PrP^231stop^-cells that express 1.8 fold higher levels of PrP^C^ compared to M17 cells. Quantitative analysis of one representative experiment is shown.

**Table 1:** Correlation between FAC induced PrP and ferritin aggregation and cytotoxicity.

Cell line	PrP aggregation	Ferritin aggregation	Condensed chromatin (%)
M17	−/+	+	18±1
PrP^C^	+	+	*31±2
**Pathogenic mutations**			
PrP^105L^	+	+	35±2
PrP^102L^	−	−/+	#44±3
PrP^187R^	+	+	#38±2
PrP^188T^	+	+	35±2
PrP^203I^	+	+	#39±1
PrP^211Q^	+	+	36±2
PrP^212P^	+	+	#38±2
PrP^217R^	+	+	#19±1
PrP^117V^	+	+	35±1
PrP^200K^	+	+	#38±2
PrP^196K^	+	+	36±2
**Non-pathogenic mutations**			
PrP^Δ51–89^	−	+	#43±2
PrP^Δ23–89^	−	+	#45±3
PrP^231stop^	−	+	#20±2
PrP^217stop^	−/+	+	#19±1

FAC: 0.1 mM for 48 hours.

n = 6 independent evaluations for all cell lines. M17 vs. PrP^C^:

*p<0.001; PrP^C^ vs. mutant cell lines:

^**#**^ *p*<0.001.

To evaluate the relationship between PrP aggregation and redox-iron induced toxicity, cells expressing PrP^C^ that aggregates, and PrP^102L^ that does not aggregate in response to FAC were exposed to 0.1 mM FAC for 48 hours, and levels of reactive oxygen species (ROS) and markers of cell death were compared (Figure 7 A–D). Immunofluorescence staining shows intracellular aggregates of PrP^C^ and minimal aggregation of PrP^102L^ under these conditions (Figure 7 A, panels 2 and 4). Estimation of cytotoxicity by Annexin V and TUNEL staining shows increased death in PrP^102L^-cells relative to similarly treated PrP^C^-cells (Figure 7 B, panel 4 vs. 1–3, and Figure 7 C, panel 2 vs. 1). In addition, PrP^102L^-cells show increased reaction for ROS when exposed to FAC (Figure 7 D, panel 4 vs. panels 1–3), suggesting impaired ability to quench FAC induced free radicals.

A comprehensive analysis of 17 different cell lines exposed to FAC for 48 hours shows maximal cytotoxicity in cells expressing non-aggregating mutant PrP forms (PrP^Δ51–89^, PrP^Δ23–89^, PrP^102L^), followed in decreasing order by aggregating mutant PrP forms (PrP^187R^, PrP^188T^, PrP^203I^, PrP^212P^, PrP^200K^), aggregating normal PrP^C^ and certain mutant PrP forms (PrP^196K^, PrP^117V^, PrP^211Q^, PrP^188T^, PrP^105L^), and the least in non-transfected M17 and cells expressing secreted PrP^C^ forms (PrP^231stop^, PrP^217stop^) (Table 1). Cells expressing PrP^217R^ are an exception since these are relatively resistant to FAC despite their tendency to aggregate [30]. The difference in cytotoxicity between different cell lines does not correlate with PrP expression levels (Figure 8 A and B).

### FAC induced toxicity is mitigated by astrocytes

**Figure 9:**
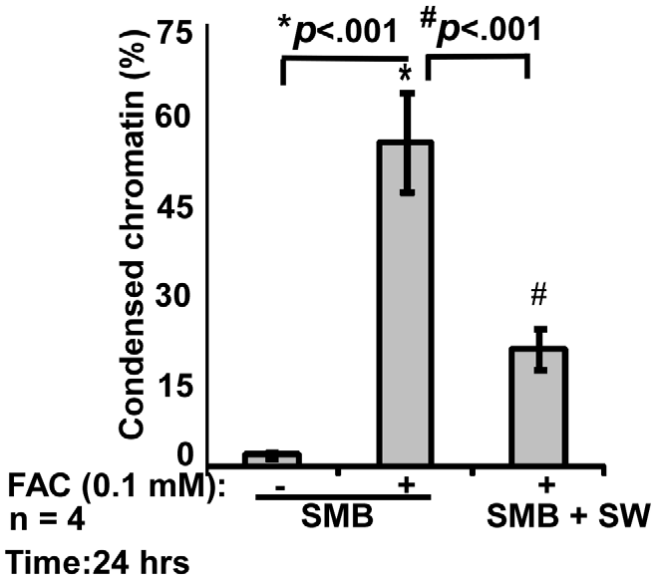
FAC induced toxicity is mitigated by astrocytes. Exposure of SMB cells to FAC causes a significant increase in toxicity that is reduced by co-culture with SW cells. *p<0.001 as compared to untreated SMB cells; ^#^ *p*<0.001 relative to FAC treated SMB cells.

Since scrapie infected cell lines ScN2a and SMB respond in a similar fashion to FAC as PrP^C^-cells [26], the relationship between PrP^Sc^-ferritin aggregates and cytotoxicity was evaluated as above. Thus, SMB cells were exposed to FAC for 24 hours, the time point at which PrP^C^-cells show an exponential increase in toxicity (Figure 3 B), and cell viability was assessed. Compared to untreated controls, exposure to FAC increases the number of dead cells from 2 to 55% (Figure 9). Surprisingly, co-culture with an astrocytoma cell line SW1088 (SW) decreases the number of dead cells to 20%, demonstrating a protective effect either by direct contact or through secretion of anti-oxidants (Figure 9).

**Figure 10:**
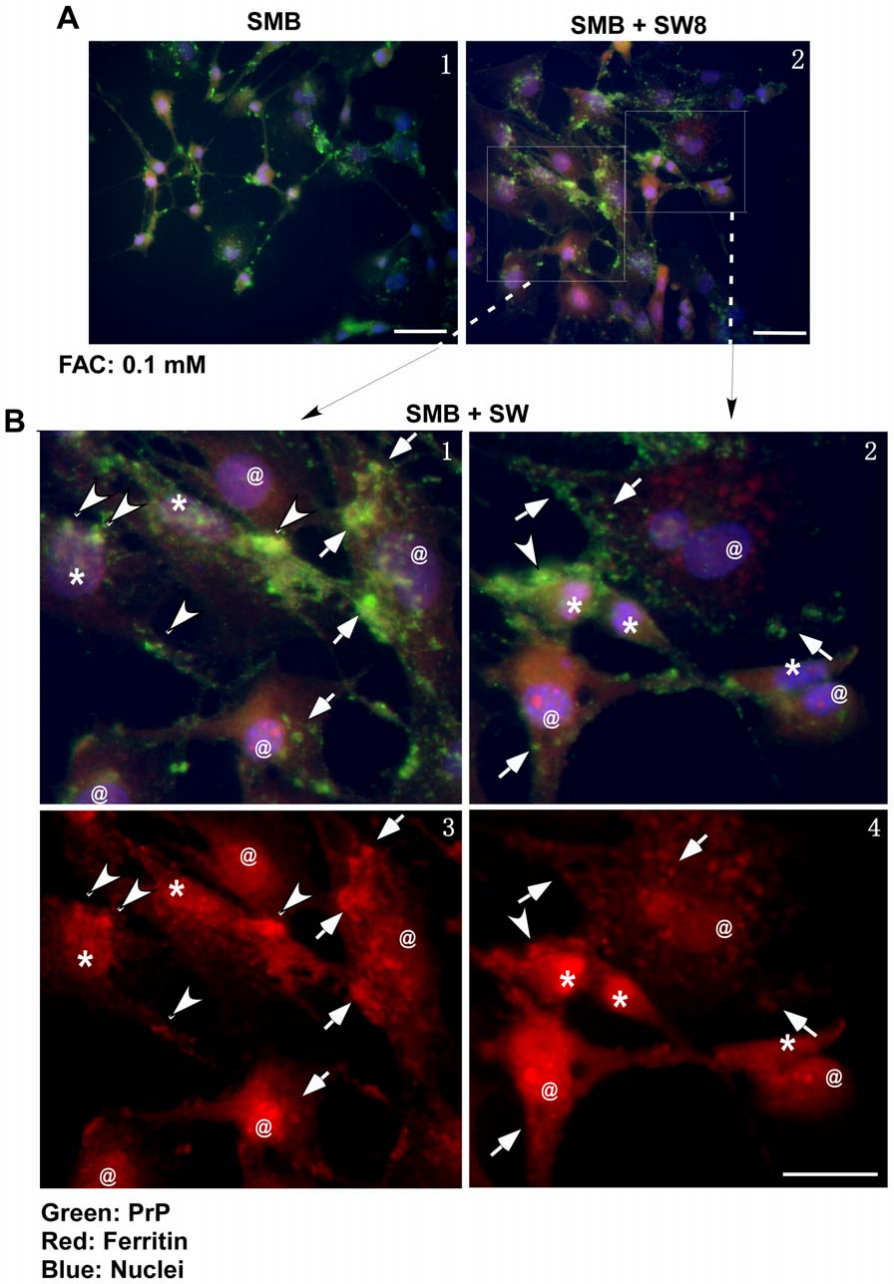
PrP-ferritin aggregates are phagocytosed by astrocytes. (**A**) SMB cells (panel 1) or co-cultures of SMB and SW cells (panel 2) were exposed to the indicated amount of FAC and immunostained for PrP (green), ferritin (red), and nuclear dye Hoechst (blue). Punctate staining of PrP is noted intracellularly and in cellular processes of SMB cells (panels 1 and 2, green). SW cells are larger in size, show minimal reactivity for PrP, and contain large nuclei with open chromatin (panel 2), allowing distinction from SMB cells. Bar: 10 µm. (**B**) Higher magnification of boxed areas from panel 2 shows reactivity for PrP within SMB cells and their processes (panels 1 and2, (*)). SW cells show minimal staining for PrP, increased reactivity for ferritin, and large nuclei with open chromatin (panels 3 and 4, (**@**)). Aggregates of PrP in SMB cells co-immunostain for ferritin (panels 1–4, arrow-heads), suggesting co-aggregation of PrP and ferritin. Aggregates of PrP and ferritin are detected on the cell surface and cytosol of SW cells, indicating phagocytosis by the latter (panels 1–4, arrows). (Only prominent groups of aggregates are marked for clarity). Bar: 10 µm.

To differentiate between these possibilities, SMB cells were co-cultured with SW cells in the presence of FAC for 24 hours and immunostained for PrP and ferritin. Cell viability was assessed by counting condensed nuclear chromatin stained with Hoechst (Figure 10 A, panels 1 and 2). SMB cells (_*_) are distinguishable from SW cells (**@**) that stain minimally for PrP (green), robustly for ferritin (red), and contain large, open nuclei (Figure 10 B, panels 1–4). FAC exposed SMB cells show prominent intracellular aggregates that co-immunostain for PrP (green) and ferritin (red) (Figure 10 B, panels 1–4, arrow-heads). Several of these aggregates appear to have undergone phagocytosis by adjacent SW cells (Figure 10 B, panels 1–4, arrows). Neither cell line shows signs of toxicity despite the presence of intracellular PrP-ferritin aggregates (Figure 10 B, panels 1–4). Similar evaluation of SMB cells in the absence of astrocytes (Figure 10 A, panel 1) or in the presence of astrocyte CM shows significant toxicity by FAC (data not shown). These observations indicate that PrP-ferritin aggregates are toxic, and elimination of these complexes by SW cells improves cell viability despite the presence of FAC.

**Figure 11:**
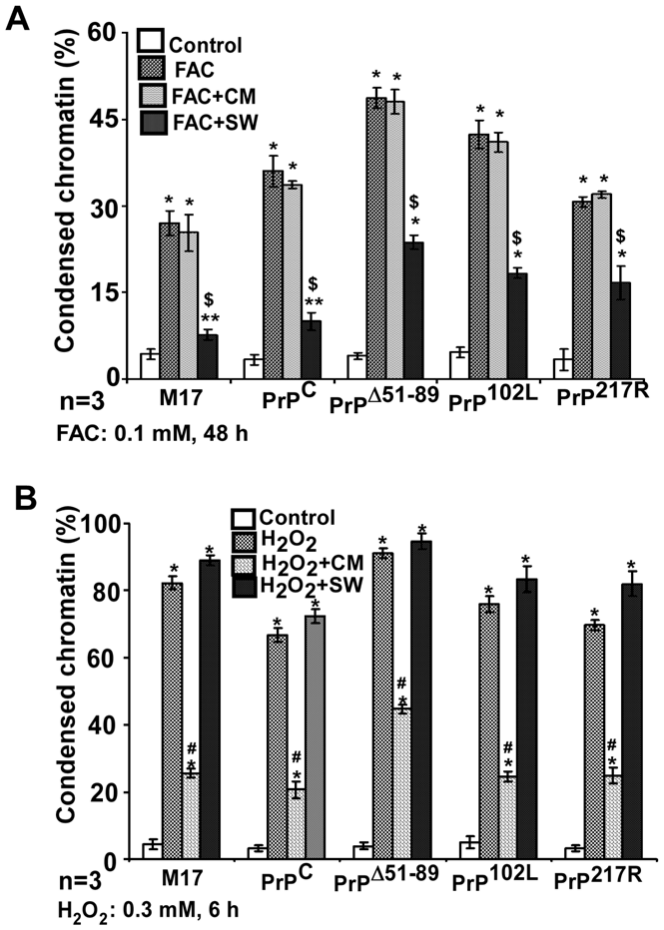
FAC induced toxicity is through PrP-ferritin aggregates. (**A**) Quantification of FAC induced cell death in M17, PrP^C^ and mutant cell lines PrP^Δ51–89^, PrP^P102L^, and PrP^217R^ when cultured in the presence of SW CM or co-cultured with SW cells shows significant toxicity by FAC in all cell lines, minimal protective effect of CM, and significant rescue by co-culture with SW cells. *p<0.001; **p<0.01 as compared to untreated controls; **^$^** *p*<0.001 as compared to FAC and FAC+CM treated cells. (**B**) Exposure of the same cell lines to H_2_O_2_ induces death in all cell lines as expected, and significant rescue by SW CM. Co-culture with SW cells has no measurable effect. *p<0.001 as compared to untreated controls; **^#^** *p*<0.001 as compared H_2_O_2_, H_2_O_2_+SW treated cells.

A similar evaluation was carried out on neuroblastoma cells expressing normal and mutant PrP forms to ascertain the generality of this phenomenon. Accordingly, PrP^C^ and mutant cell lines PrP^Δ51–89^, PrP^P102L^, and PrP^217R^ were cultured under four different conditions: 1) normal medium (controls), 2) medium supplemented with 0.1 mM FAC, 3) 1∶1 mixture of fresh and SW-CM supplemented with 0.1 mM FAC, and 4) 1∶1 mixture of a specific cell line and SW cells in medium supplemented with 0.1 mM FAC. Cells were cultured for 48 hours, and viability was assessed by counting Hoechst stained condensed nuclear chromatin. As noted for SMB cells, FAC induces significant toxicity in all cell lines tested. Addition of CM does not alter cell viability, whereas co-culture with SW cells reduces cell death significantly. Quantitative comparison shows that co-culture with SW cells decreases FAC induced toxicity by 74%, 72%, 51%, 57% and 46% in M17, PrP^C^, PrP^Δ51–89^, PrP^P102L^, and PrP^217R^ -cells respectively (Figure 11 A).

Exposure of the same cell lines to H_2_O_2_, another source of free radicals, shows different results. All cell lines are very sensitive to 0.3 mM H_2_O_2_ for 6 hours, reaching near 100% cell death. Co-culture with SW cells has no protective effect, whereas culture in astrocyte CM decreases H_2_O_2_ induced toxicity by 70%, 69%, 51%, 68% and 64% in M17, PrP^C^, PrP^Δ51–89^, PrP^P102L^, and PrP^217R^-cells respectively (Figure 11 B). Together, the above results demonstrate that FAC induced toxicity is mediated by intracellular PrP-ferritin aggregates, while H_2_O_2_ induces toxicity through extracellular free radicals.

## Discussion

This report elucidates possible mechanism(s) underlying iron mediated neurotoxicity in prion disorders, and clarifies the role of PrP and astrocytes in this process. Using cell models of familial prion disorders, we demonstrate that aggregation of PrP^C^ and certain mutant forms of PrP in response to redox-iron protects cells from iron-induced acute toxicity. However, subsequent co-aggregation of PrP with ferritin renders these aggregates redox-active, resulting in an exponential increase in cytotoxicity at later time points. Mutant PrP forms that do not aggregate under similar conditions are not protective, leading to acute cell death. In addition, astrocyte mediated phagocytosis and perhaps autophagy of PrP-ferritin aggregates protects cells against toxicity, highlighting specific cell types and biochemical processes that influence neuronal survival. Mouse scrapie infected cell lines and prion disease affected hamster and human brains show similar results, demonstrating the relevance of these findings to prion disease associated neurotoxicity.

Redox-iron has emerged as an important cause of neurotoxicity in several neurodegenerative conditions in addition to prion disorders, including Alzheimer's disease, Parkinson's disease, Huntington's disease, Friedreich's ataxia, multiple sclerosis, Pick's disease, Hallervorden-Spatz disease, tardive dyskinesia, aceruloplasminemia, and others [31]–[37]. In each of these disorders, a redox-active metal such as iron is reduced in the presence of a specific protein, resulting in the generation of free radicals and consequent aggregation of the protein [36], [38]–[40]. The pathophysiology of prion disorders renders affected brains especially prone to metal dyshomeostasis since PrP^C^ is involved in the uptake and transport of iron and copper [18], [20], [26], and aggregation to the PrP^Sc^ form is likely to alter copper and iron homeostasis due to the combined effect of loss of PrP^C^ function and sequestration of these metals by PrP^Sc^, rendering the aggregates redox-active. A specific example of this phenomenon has been reported in a cell model, demonstrating the sensitivity of PrP^C^ to excess redox-iron [25]. However, the molecular mechanism(s) underlying redox-iron mediated neurotoxicity and its role in the pathogenesis of familial prion disorders has not been explored.

Our results show that cells expressing normal and mutant forms of PrP elicit a distinct response when exposed to a source of redox-iron such as FAC. M17 cells that over-express PrP^C^ show resistance to higher amounts of FAC relative to non-transfected cells, suggesting a dose-dependent protection against redox-iron. Surprisingly, PrP^C^-cells show significantly higher toxicity relative to M17 cells after the first 16 hours of exposure even if FAC is removed from the medium (unpublished observations). One likely explanation for these observations is that interaction of redox-iron with copper and iron bound PrP initiates the Fenton reaction, resulting in denaturation and aggregation of PrP at the cell surface [35], [41]–[43]. Since this reaction utilizes free radicals, initial aggregation of PrP provides protection against redox-iron induced toxicity. However, subsequent transport of PrP aggregates to lysosomes and association with ferritin results in the formation of a complex that is resistant to degradation by proteases and is itself redox-active [25, this report], causing an exponential increase in cell death. Since M17 cells express lower levels of PrP^C^, a similar paradoxical response to redox-iron does not occur. Morphological evaluation of FAC exposed PrP^C^ cells confirms the accumulation of PrP-ferritin complexes in lysosomes, and EM analysis indicates that aggregated ferritin is rich in iron, some of which precipitates out and is visible as electron dense granules. It is likely that the iron-rich nature of these aggregates combined with resistance to proteolytic digestion is responsible for the late toxicity in PrP^C^ cells.

Cells expressing mutant PrP forms show significantly higher toxicity to FAC than PrP^C^-cells at all time points tested. Since several of the mutant PrP forms aggregate with similar kinetics as PrP^C^, it is likely that mutant PrP is inefficient in sequestering free radicals at the cell surface and intracellularly, resulting in direct damage from exogenous redox-iron and from intra-cellular redox-active mutant PrP-ferritin aggregates. Certain fragments of mutant PrP forms generate significantly higher levels of free radicals when exposed to copper, supporting this claim [39], [44]–[46]. It is surprising to note that cells expressing PrP^217R^ show higher toxicity than PrP^C^ cells in the first 16 hours, followed by a drop in cell death after 48 hours. It is likely that PrP^217R^ aggregates are not effective in quenching free radicals on the cell surface, but form fairly stable intracellular aggregates with ferritin that are less redox-active than PrP^C^-ferritin, thereby alleviating late toxicity. Cells expressing anchor-less PrP forms such as PrP^231stop^ and PrP^217stop^ show similar toxicity as M17-cells, indicating that expression of PrP on the plasma membrane is necessary for protection, and intra-cellular aggregation of PrP-ferritin complexes is essential for late toxicity. Cells expressing other mutations such as PrP^102L^, PrP^Δ23–89^, and PrP^Δ51–89^ that do not aggregate in the presence of FAC show increased levels of intracellular ROS and toxicity at all time points tested. The extent of cell death in these cells is significantly higher at all time points than PrP^C^ and mutant cell lines that aggregate in response to FAC, supporting the protective role of PrP aggregation against acute toxicity. Scrapie infected cell lines show significantly higher toxicity to FAC such that ten times lower levels are sufficient to cause apoptosis (unpublished observations). Increased sensitivity of these cells to FAC may be due to lower expression of PrP^C^ on the cell surface combined with relatively rapid accumulation of PrP^Sc^-ferritin aggregates on pre-existing PrP^Sc^ seed.

It is surprising that PrP^102L^ and PrP^105L^ that segregate with GSS respond differently to FAC despite their physical proximity within the protein sequence and similar amino acid mutations. Although information on the copper and iron binding capacity of PrP^102L^ and PrP^105L^ is not available, it is known that PrP^102L^ shows delayed recycling to the plasma membrane [47], a defect that may be responsible for increased accumulation of iron in cellular ferritin in PrP^102L^ cells and a paradoxical phenotype of iron deficiency [19]. Perhaps internalized PrP^102L^ forms a complex with ferritin that interferes with its transport back to the plasma membrane, accounting for the lack of free radical quenching activity of PrP^102L^ on the cell surface, absence of PrP^102L^ aggregates, increased levels of intracellular ROS, and increased susceptibility to FAC. Failure of PrP^Δ23–89^ and PrP^Δ51–89^ to aggregate is perhaps due to decreased iron and/or copper binding, compromising their ability to protect cells against FAC induced injury.

All the cell lines tested except PrP^102L^ show up regulation and aggregation of ferritin in response to FAC. Immunofluorescence evaluation demonstrates accumulation of ferritin in lysosomal structures in association with aggregated PrP, and EM analysis of FAC exposed PrP^C^ cells reveals accumulation of electron-dense, iron-rich aggregates in structures consistent with lysosomes/autophagosomes. Biochemical analysis of FAC exposed PrP^C^ cells confirms this observation by demonstrating up regulation of LC3-II, a marker of autophagy [27]–[29]. A similar increase in LC3-II levels is observed in scrapie infected hamster and sCJD affected human brains, demonstrating the occurrence of similar processes in prion disease affected brains. In hamster samples, levels of LC3-II increase with disease progression, suggesting an increase in autophagosome activity with disease progression. Whether this phenomenon occurs due to accumulation of PrP^Sc^-ferritin complexes is unclear from our data, but is a likely possibility [19], [28].

Our observations on FAC exposed scrapie-infected cell lines show that co-culture with astrocytoma cells induces active phagocytosis of PrP^Sc^-ferritin complexes and rescues cells from toxicity. Culture in astrocyte conditioned medium is not protective, suggesting that protection is derived from clearance of toxic aggregates by phagocytosis, not by quenching free radicals in the extra-cellular milieu. This assumption is supported by the fact that co-culture of FAC exposed scrapie infected cells or cell lines expressing normal and mutant PrP forms does not protect cells from H_2_O_2_ induced toxicity, whereas culture in astrocyte CM reduces toxicity by H_2_O_2_ significantly. These observations suggest that PrP-ferritin aggregates are intrinsically toxic, perhaps due to their redox-active nature. Although extracellular FAC could induce toxicity due to free radicals independent of PrP-ferritin aggregates, the concentrations of FAC used in our experimental paradigm are non-toxic for at least 48 hours, suggesting that toxicity is mainly through intracellular events. It is likely that within the brain astrocytes protect scrapie infected neurons by phagocytosing PrP^Sc^ aggregates, thereby reducing free radicals in the intra- and extra-cellular milieu.

In conclusion, this report highlights the paradoxical role of PrP in redox-iron induced toxicity, and escalation of the toxic signal by mutant PrP forms. Since chelation of iron from diseased brain homogenates reduces the redox-activity and protease-resistance of PrP^Sc^ complexes [25], it is possible that restoration of iron homeostasis in diseased brains would decrease or eliminate both extra-cellular toxicity by redox-iron and intracellular toxicity by redox-active PrP^Sc^-ferritin complexes. Although encouraging, restoration of iron homeostasis in diseased brains is a daunting task due to the complexity of biochemical pathways and cellular interactions responsible for this process. The protective effect of phagocytosis is encouraging, but further investigations are necessary to fully understand and exploit this pathway.

## Materials and Methods

### Materials and antibodies

Primary antibodies 3F4 and 8H4 against PrP were purchased from Signet Laboratories (Dedham, MA) and provided by Drs. Pierluigi Gambetti and Man Sun-Sy (National Prion Surveillance Center, Case Western Reserve University). LC3 antibody was obtained from Cell Signaling Technology (Cat. No. 2775). Polyclonal anti-ferritin antibody was procured from Sigma-Aldrich (St. Louis, MO, Cat. No. F5012), horseradish peroxidase (HRP)-labeled secondary antibodies were from GE Healthcare (Little Chalfont, Buckinghamshire, United Kingdom), FITC (fluorescein isothiocyanate) and TRITC (tetramethylrhodamine B isothiocyanate) labeled secondary antibodies were from Southern Biotechnology Associates (Brimingham, AL). Hoechst was obtained from Invitrogen (Cat. No. H3570), and Hygromycin was procured from Calbiochem (San Diego, CA, Cat. No. 400051). All other reagents including Ferric Ammonium Citrate (FAC) were obtained from Sigma-Aldrich, unless otherwise mentioned.

### Cell culture

Human neuroblastoma cells (M17) over expressing PrP^C^ or mutant PrP forms were generated and cultured as described earlier [30]. Additional point mutations were generated for this study using the QuickChange XL Site-Directed Mutagenesis Kit (Agilent Technologies, La Jolla, CA 92037, Cat. No. 200517) and maintained in the presence of hygromycin as described previously [48]. Scrapie-infected mouse neuroblastoma cells (ScN2a) and SMB cells were obtained from Dr. Byron Caughey (Rocky Mountain Laboratories) and Dr. Glenn Telling (University of Kentucky) respectively, and maintained in Medium 199 supplemented with 10% Normal Calf Serum (NCS), and 1% Penicillin-Streptomycin (PS). Human astrocytoma cell line SW1088 (ATCC No. HTB-12) was obtained from American Type Culture Collection (ATCC, Manassas, VA) and cultured in high glucose DMEM supplemented with 10% FBS and 1% PS. All cultures were maintained at 37°C in a humidified atmosphere containing 5% CO_2_.

### Aggregation assay

Equal numbers of cells expressing PrP^C^ or a specific mutant PrP form were cultured in complete medium in the presence of 0.1 mM FAC dissolved in Opti-MEM. Control cultures were maintained under similar conditions with no added FAC. Following an incubation of 48 hours, cells were checked for any signs of toxicity, rinsed with cold PBS, and lysed in a buffer containing 100 mM NaCl, 20 mM Tris-HCl, pH 7.4, 1% NP-40, 0.5% DOC and 10 mM EDTA. Lysates were subjected to differential centrifugation to identify aggregated PrP forms as described in earlier reports [25]. In short, following a low speed spin at 290 g to isolate low speed supernatant (S1) and pellet fractions (P1), the S1 fraction was subjected to ultra-centrifugation at 100,000 g to separate the high speed supernatant (S2) and aggregated pellet (P2) fractions. Proteins from each fraction were precipitated with cold methanol, boiled in sample buffer, and subjected to Western blot analysis. Transferred proteins were probed for PrP and ferritin using specific antibodies.

### Western blot analysis

Cell lysates or 10% brain homogenates prepared in lysis buffer as above were resolved on SDS-PAGE and transferred to a PVDF membrane. After blocking the membrane with 5% milk in TBST (20 mM Tris-HCl, pH 7.4, 150 mM NaCl, and 0.1% Tween 20), membranes were washed with TBST and probed with specific antibodies followed by HRP-conjugated secondary antibodies. Reactive bands were visualized using ECL detection kit (Amersham Biosciences Inc.).

### Immunostaining

ScN2a or SMB cells cultured on poly-L-lysine coated cover slips were exposed to FAC for 24 hours and processed for immunostaining. For co-culture experiments, equal number of SMB and SW1088 cells were cultured on coverslips, exposed to 0.1 mM FAC for 24 hours, and processed for immunostaining using PrP and ferritin specific antibodies as described previously [30].

### Co-immunoprecipitation

Cells expressing PrP^C^ and specific mutant PrP forms cultured as above were exposed to 0, 0.05, and 0.1 mM FAC for 48 hours and lysed in a buffer containing 2% Triton X-100 in Tris-buffered saline (20 mM Tris-HCl, pH 7.4, 150 mM NaCl,) containing protease inhibitor cocktail. Cell debris was sedimented by centrifugation at 800 g, and clarified supernatants were rocked with 4 µl of 8H4 or 2 µl of anti-ferritin antibody in presence of 1% bovine serum albumin and 0.1% *N-*lauryl sarcosine. After an overnight incubation at 4°C, antigen-antibody complexes were captured with protein A agarose beads (Roche, Cat. No. 1134515) and washed extensively with wash buffer (150 mM NaCl, 10 mM Tris-HCl, pH 7.8, 0.1% *N-*lauryl sarcosine and 0.1 mM phenylmethylsulfonyl fluoride). Bound proteins were eluted by boiling in sample buffer (125 mM Tris-HCl, pH 6.8, 3% SDS, 10% glycerol and 5% β-mercaptoethanol), resolved by SDS-PAGE, and subjected to immunoblotting with anti-PrP antibody 3F4.

### Electron Microscopy

Cells grown in the presence or absence of 0.1 mM FAC were fixed in 4% paraformaldehyde in 0.2 M PHEM buffer (120 mM PIPES, 50 mM HEPES, 4 mM MgCl_2_ and 20 mM EGTA) for 24 h. Fixed cells were washed twice with PBS+ (PBS containing 0.15 M glycine) and incubated with 1% gelatin for 30 min at 37°C. After washing with PBS, cells were stored in storage solution (0.1 M PHEM buffer containing 4% paraformaldehyde). For electron microscopy, cells were refixed in PBS containing 2.5% glutaraldehyde, 2% paraformaldehyde, and 4% sucrose for 2 hours followed by 1% osmium tetroxide for 1 hour. After dehydrating in increasing concentrations of ethanol, cells were embedded in Epon 812 and examined using a JEOL 1200EX electron microscope.

### Toxicity assays

To estimate toxicity of FAC or H_2_O_2_, scrapie infected cell lines ScN2a and SMB or PrP^C^ and mutant cell lines were exposed to 0, 0.05 or 0.1 mM FAC for 6–48 hours and analyzed for cell death by DNA fragmentation, LDH release and TUNEL assays, staining with Annexin-V, and by calculating percentage of Hoechst stained condensed nuclei in 20 different 40x microscopic fields. For cells treated with FAC, LDH assay could not be performed as it has been reported that bipyridyl derivatives of divalent metal ions inhibit LDH. [49].

For LDH assay, PrP^C^ and mutant cell lines cultured in 24 well plates were exposed to different concentrations of H_2_O_2_ for the indicated times, and release of LDH was assessed using the LDH-cytotoxic assay kit from Wako (cat. No. 299–50601). Based on these evaluations, optimum concentrations of H_2_O_2_ and the time of exposure were decided. FAC concentration and time of exposure was decided as reported earlier [25]. DNA fragmentation assay was carried out on control and FAC exposed cells after 6 hours. In short, cells were rinsed with PBS and lysed in a buffer containing 10 mM Tris-HCl, pH 7.5, 0.25% NP40, RNAase A (2 mg/ml), and proteinase-K (2 mg/ml). After incubating the mixture for 20 minutes at 37°C, lysates were centrifuged at 14,500 rpm for 15 minutes and supernatant was analyzed on 1.5% agarose gel. DNA ladder was visualized by staining with ethidium bromide. Tunnel assay was carried out using the *In situ* Cell Death detection Kit, TMR red (Roche, Germany). Staining for Annexin V was performed on cells cultured on poly-L-lysine coated coverslips using the Annexin V conjugate (Molecular Probes, Inc., Cat # A13199). TUNEL positive and Annexin V positive cells were examined under a fluorescence microscope. The level of ROS produced within control and treated cells was measured by the cell permeable, non-polar, H_2_O_2_-sensitive probe 5,6-chloromethyl-20,70 dichlorodihydro-fluorescein-diacetate (CM-H2DCFDA) from Sigma, USA. Cells cultured on poly-L Lysine coated coverslips were exposed to different experimental conditions and treated with 5 µM solution of CM-H2DCFDA at room temperature for 45 minutes. Cells were then washed with ice-cold PBS and fluorescence intensity of intracellular DCFDA was observed under the microscope.

### Protection assay

Equal number of SMB and SW1088 cells were seeded to achieve 70% confluence on poly-L-lysine coated cover slips and cultured overnight in DMEM supplemented with 10% FBS and 1% PS. Cultures were examined under the microscope to make sure the cells are making physical contact with adjacent cells. The medium was removed, and fresh medium supplemented with 0.1 mM FAC was added. After further culture for 24 hours, cells were rinsed with cold PBS, fixed with paraformaldehyde, and immunostained for PrP and ferritin as described before [30]. For cell lines expressing PrP^C^ and mutant PrP forms, four different experimental paradigms were tested in the presence of 0.1 mM FAC for 48 hour and 0.3 mM H_2_O_2_ for 6 hours: 1) control cultures with normal medium, 2) cultures supplemented with FAC or H_2_O_2_, 3) addition of FAC or H_2_O_2_ in a 1∶1 mixture of fresh medium and conditioned medium (CM) collected from SW1088 cultures grown to near confluency and clarified by centrifugation, and 4) co-culture of a 1∶1 mixture of the specific cell line and SW1088 cells. At the end of each incubation, cells were rinsed with cold PBS, fixed with paraformaldehyde, and nuclei were stained with Hoechst. Triplicate coverslips from each condition were examined and the percentage of condensed nuclei was calculated from 20 different random fields examined under a 40x lens.

### Statistical analysis

All experiments were repeated at least three times. The results are expressed as mean ± standard error of mean (SEM). Statistical analysis was done by unpaired Student's t-test when comparing two groups. For multiple groups, one way ANOVA followed by Bonferroni multiple comparison post hoc test was done using GraphPad Prism software (Version 4.03, GraphPad Inc., San Diego, CA, USA). Differences were considered significant at *p*<0.05.

## Supporting Information

Figure S1 Differential fractionation of proteins is not an artifact of loading. (A & B) PVDF membranes used for probing PrP and ferritin in Figure 1 were stained with Ponceau S to visualize all transferred proteins. Comparison of protein loading between different S1 and S2 fractions and P1 and P2 fractions is similar in FAC exposed and control samples for all cell lines.
(1.97 MB TIF)
