# Supplementary figures and images for: Paradoxical Role of Prion Protein Aggregates in Redox-Iron Induced Toxicity
Source: PLoS One. 2010 Jul 6;5(7):e11420. doi: 10.1371/journal.pone.0011420 (PMC2897850; doi:10.1371/journal.pone.0011420)

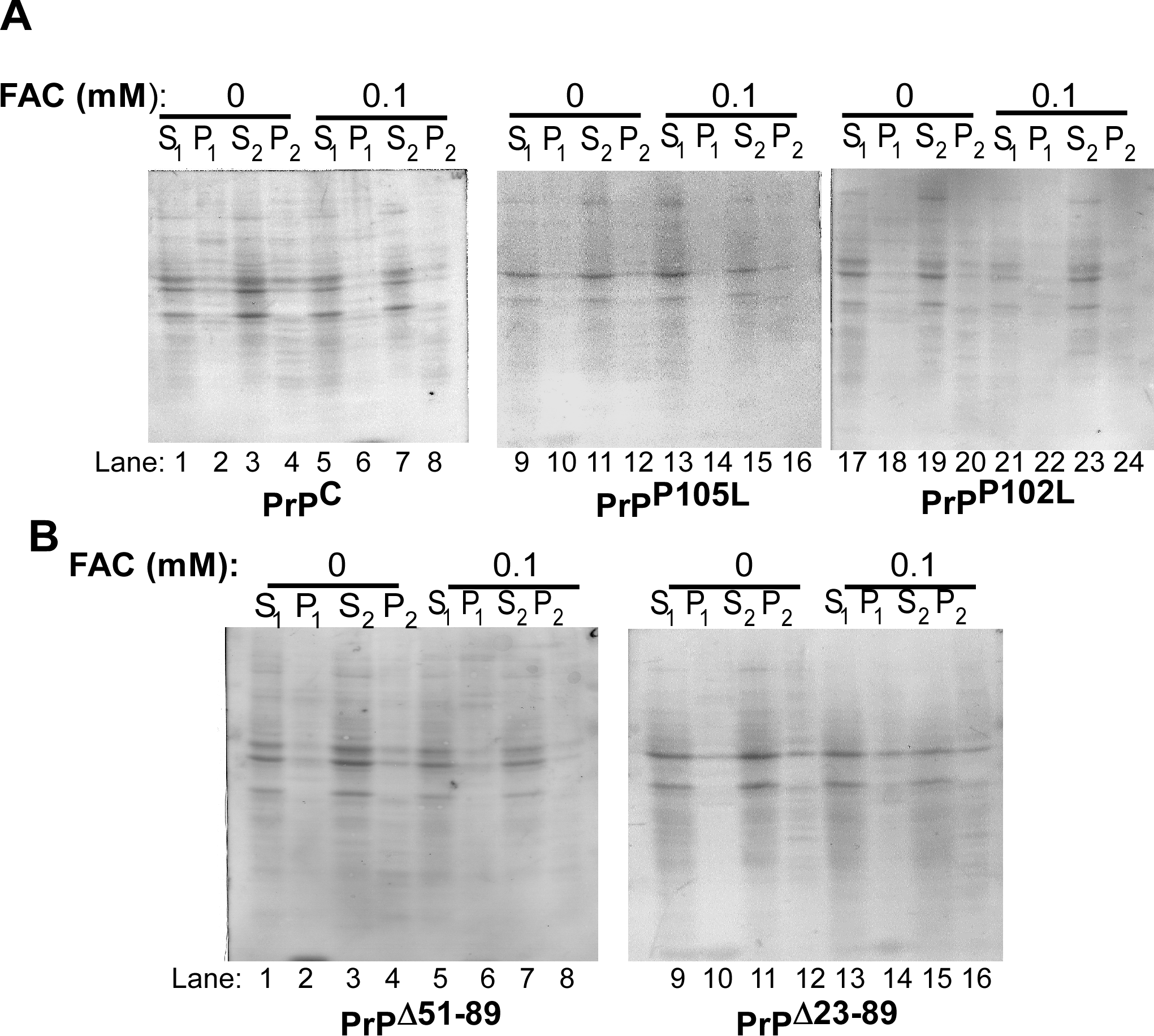

Supplement: Figure S1 — Differential fractionation of proteins is not an artifact of loading. (A & B) PVDF membranes used for probing PrP and ferritin in Figure 1 were stained with Ponceau S to visualize all transferred proteins. Comparison of protein loading between different S1 and S2 fractions and P1 and P2 fractions is similar in FAC exposed and control samples for all cell lines. (1.97 MB TIF) [file pone.0011420.s001.tif]
